# “Take Me Back to My Homeland Dead or Alive!”: The Myth of Return Among London’s Turkish-Speaking Community

**DOI:** 10.3389/fsoc.2021.630558

**Published:** 2021-03-16

**Authors:** Mustafa Cakmak

**Affiliations:** Sociology Department, Keele University, Keele, United Kingdom

**Keywords:** Turkish-speaking community, diaspora, myth of return, return visits, mundane pilgrimage

## Abstract

In classical diaspora literature, the “myth of return” has major significance. It is believed that the “myth of return” is embedded in the minds of immigrants from their arrival. This paper examines post-migration mobilities of the Turkish-speaking community in North London as well as the shift in narratives of homeland among diaspora communities; from the “myth of return” to ritual-like visits or mundane pilgrimages. My ethnographic study analyses the post-migration homeland visiting patterns of the Turkish-speaking community into four categories. I discuss how narratives of episodic homeland visits and the desire to be buried in the homeland have replaced the myth of return.

## Introduction

In classical diaspora literature, the “myth of return” has a major significance. It is believed that the “myth of return” is embedded in the minds of immigrants from their arrival. What is known as the “myth of return” (Anwar, 1979) refers to more than a demographic movement. In sociological and anthropological literature, while there are nuances, almost all immigrant communities are portrayed as people motivated by the idea of return whilst simultaneously struggling to maintain links with their homeland (Dahya, 1973; Jeffery, 1976; Anwar, 1979; Robinson, 1986; Shaw, 1988; Van Hear, 1998; Levitt, 2003; Cohen, 2008; Cetin, 2016; Eylem et al., 2016). This is highlighted by Dahya (1973), who claims that the myth of return acts as a cohesive force with the purpose being consolidation of the kinship boundaries of the community and links with their homeland. It is the emotional tie that diasporic communities keep with their home countries around which they renegotiate their identity. From this perspective, diaspora researchers have long discussed diasporic communities’ narratives in exile which are centered around the concept of home and return. However, as Hall (1990) argues, diasporic identities are not static identities that can only be preserved with the idea of a return.

Diaspora does not refer us to those scattered tribes whose identity can only be secured in relation to some sacred homeland to which they must at all costs return, even if it means pushing other peoples into the sea. (…) Diaspora identities are those which are constantly producing and reproducing themselves anew, through transformation and difference. (Hall, 1990: 235)

Although the theory is labeled a myth because it is not necessarily about feasibility of return, my research findings suggest not only a lack of plans to return but also a shift of the narratives of homeland which are no longer centered around the myth of return. Lie (1995:304) states that “it is no longer assumed that immigrants make a sharp break from their homelands”. Some of them travel regularly; some of them travel back and forth and/or engage in transnational working relationships while living abroad. The second and third generations, who do not have any first-hand experience or memory of their ancestral homeland have received their primary socialization from a “host” country and build a sense of having multiple homes with these visits (Al-Ali and Koser, 2002). I argue that narratives of return are instead replaced by regular visits to the homeland. It is the time spent in diaspora alongside changes in their homeland that changes the narrative of their homeland. By analyzing post-migration mobilities of the Turkish-speaking community in London and discussing the meaning of the findings in light of classic and contemporary diaspora theories, I seek to provide a broader and deeper understanding of the construction of identity in the community.

## Methodology

I conducted an ethnographic study to understand how members of the Turkish-speaking community in North London perform their identity through cultural practices in everyday life. My research questions how identity is constituted and maintained in a diasporic environment and asks: how do members of the diaspora negotiate between home and host cultures; how do they reinterpret the cultural landscape to perform identities; and how does collective memory and narration of the past affect the younger generation’s sense of belonging?

In order to answer these questions, I conducted ethnographic research which comprised of several methods; 10 months of ethnographic fieldwork (between September 23, 2015 and July 23, 2016), visiting coffee houses, off-licenses, kebab shops, community centers, mosques and assessing various aspects of cultural life of the community. Also, during three years of my Ph.D. research I strolled the streets of North London as a *flâneur* living within the research environment. I observed everyday life and analyzed visual materials and their relation to place and identity. In addition to observation, I also used oral history to gather experiences and memories of the community. Most members of the community were willing to talk, so I used this opportunity strategically and wrote anonymously the anecdotes or stories narrated spontaneously. I also recruited some of the participants for in-depth interviews, which I conducted at their work spaces or third places. In total, I conducted interviews with twenty-nine people. Fourteen of the interviewees were first-generation immigrants, twelve of them second-generation immigrants, and three of them third generation immigrants. Nine of the participants were from the first wave of Turkish migration, five of them from the second wave, eleven of them from the third wave and four of them from the fourth wave. Overall, eight of the research participants were Turkish Cypriot, eleven of them were Turkish and ten of them were Kurdish. With this sampling, I aimed to avoid underrepresentation of any group.

The selection criteria is based on the objectives of the research and involvement/interaction with British culture. Thus, only those members of the community who have been living in the United Kingdom for at least ten years were invited to take part in interviews however others were included in the participant observations. In addition, I photographed home decorations and the clothes of my subjects as well as recording videos of cultural practices, events and rituals in order to understand the use of cultural materials in identity performances. By adopting this broad qualitative empirical approach, I observed the subjects in everyday settings sometimes as a tourist or as a *flaneur,* but always as a fellow member of the Turkish-speaking community.

## The Myth of Return Among Turkish-Speaking Community

My research findings suggest two main motivations for the migration of Turkish-speaking people to the United Kingdom; economic and political. According to this data, almost all of them came to the United Kingdom for a limited period in their mind with the purpose of returning to their home country like other diaspora communities. For the economically motivated group the primary purpose of moving to the United Kingdom was to save enough money to build a better life back in their home country. Therefore, they keep the “myth of return” alive both as a motivation to work abroad and to reconstruct their cultural identity. The politically motivated groups came to stay until ethnic and political tension in their home country has reduced. They have a more romantic view of their home countries as they had to leave from there, so they see it as a lost land, and they view the “myth of return” as a sacred desire in diaspora (Watson, 1977; Safran, 1991; Cohen, 1996). Economic migrants’ departure from their home country is not always purely voluntary such as in times of economic recession, yet it does not necessarily mean their life is under direct threat like political groups who face threats during a military coup, revolution or civil war. Political refugees in the United Kingdom are generally alienated in their country of origin after a political change or a civil war. All fourteen of my first-generation participants narrated that their country of origin has changed since they left, this is generally a negative change from their point of view. What they desire to return to is not the contemporary state of their home country. Instead, they wish for a nostalgic version of their homeland or a utopian future where the political change they wished for has been achieved. People from both categories are unwilling to move forward from their versions of the homeland.

Hall, (1990) argues that there is not any return to the country of origin or roots because diasporic subjects are not able to return. Among the Turkish-speaking community, except for some political subjects, the inability to return home country is not because of legal restrictions, but lifestyle choices. For example, their home country has changed since they left, and/or they personally have changed and they have become accustomed to the lifestyle in London. During our interviews and conversations, most of my research participants explained that either they or their parents had come to the United Kingdom for a limited period of time in their mind but they then decided to stay to provide a better future for their children. Another narrative expressed was that they feel that they cannot fit into the system in Turkey or Cyprus any more.

The community I grew up in is a community of Turkish people who came here, struggling to make money and go back. So, there was a deep sense of having to escape from this country (United Kingdom) as fast as they could. So, there was not a lot of joy in that. (…) Although they are trying to extract themselves from England and go back to Turkey they very much hold on to the ‘but we need to live for our children’ narrative. (Mary, 45, Second-generation, First Wave, Turkish)

As Mary narrated, the first-generation of the first migration wave from Turkey and Cyprus consisted of economic migrants who came to the United Kingdom in order to save up money to return and invest in their home countries. She is skeptical of the first wave migrants’ claim that they remained in the United Kingdom to provide better opportunities for their children. She believes that people stayed because they became accustomed to life in London and prefer it over their home country. This discussion also suggests the narrative of the myth of return fading away from the diaspora discourse.

Even the political refugees who came to the United Kingdom with the idea of going back to their home country once, the political situation became more stable and kept the romantic view about *memleket* (homeland) as a lost land, decided to remain in London. They participate in political activities in diaspora to make an impact on the political sphere in their home countries and change it from abroad in a way they desire (Cakmak, 2018). However, the narrative of the myth of return among this group also has shifted.

Because when we first came to this country, we were saving every penny because we were going to go back. (…) Because my parents are *Alevis* and they just had to sort of hide it and they didn't feel safe, and that's why they started to come here for work so they could make money and have enough capital to buy themselves a house in Mersin to hide there. And then they thought this place [London] is safer and they stayed. (Ela, 37, First-generation, Third Wave, Kurdish)

Ela and all other *Alevi* (a religious minority group in Turkey) participants narrated to me the security concerns of *Alevi* diaspora as a main motivation not to return to Turkey. Unlike previous works about diaspora communities, my research does not suggest any existing debates about the myth of return despite the relative safety in their home country.

I am not planning to spend the rest of my life in this country; I do not think I will waste it here. But I am not planning to move to Turkey in the near future (…) We will die sooner or later, better to die over there, isn’t it? North London is like a second refugee camp. (Rojda, 27, Second-generation, Third Wave, Kurdish)

Leaving behind their country of origin places refugees in a condition of “social nakedness”. This precarious situation could be described with its undefined social status, rights and responsibilities (Bauman, 2002). As Agamben (1998) argues, refugees are like “*a werewolf*” neither a beast nor a man, an outlaw that can be exposed to violence without facing legal sanctions. Refugee camps are a biopolitical zone of indistinction, refugees are banned and excluded from society (Diken, 2004). Despite living in London most of her life, Rojda, a Kurdish political activist, defined the ethnic neighborhoods in North London as a second refugee camp where the community is excluded from society and forced to survive in a state of incarceration for a lifetime. Despite her political motivations and emotional attachment to the Kurdish land, her narrative did not suggest the myth of return. She wants to keep the idea of a homeland alive because she does not want to permanently settle down in the United Kingdom. The simile of refugee camp refers to a temporary residence in diaspora until she reaches her destination; the imagined Kurdistan. Instead of discussing returning home to live there or to change it, her narrative was focused on eventually returning to the homeland to die there. Although stories of fellow diasporans moving back to their home countries and settling down successfully were narrated to me during my field study, it is not very common among the community (Keles, 2016). In addition, some of the attempts at return among diasporic subjects resulted with them moving back or returning to London. Some of my research participants told me about their stories of failed attempts to settle down in their country of origin and “returning” to London. Narratives referred to both processes of moving to Turkey to settle down and coming back to the United Kingdom as a “return” which indicates their sense of belonging to multiple homes and a feeling of “in-betweenness” (Bhabha, 1994). Therefore, adopting Peeren, (2006) chronotopic approach while discussing diaspora would be more accurate. In this approach, “home” is not perceived as left behind, static, pure or untouched. The theory of chronotope discusses diasporic identities as a multi-dimensional notion instead of just focusing on a static home and host that can be gained or lost. There are multiple sites within and transcending home country and adopted home.

I moved to home, Turkey, for ten years. I started a business there. In the 2010 crisis, I lost like 6 million pounds. I had to sell up everything to pay off my debts. And then I wanted to come to home, London. (Mary, 45, Second-generation, First Wave, Turkish).

Mary had moved to Turkey from London to re-adopt her ancestral homeland. Yet after spending 10 years there she returned to London which she considers her “actual” home. I argue that she has multiple homes representing her multiple identities. For the second-generation diaspora members, moving to Turkey or Cyprus is akin to achieving their parents’ plans. However, for the third-generation, settling down in Turkey or Cyprus is fulfilling their ancestors’ prophecies by “returning” to the “promised land” after the *exodus*. Younger generations are not familiar with institutions or social norms in Turkey or Cyprus. It is in fact not a return but moving to a foreign landscape and uniting with a society with which they have very little in common. Mary is not the only research participant who narrated to me about the failed attempt of moving to Turkey among the second-generations. Some other participants of younger generations told me that they considered moving to Turkey or Cyprus at certain points in their life and then decided against it. Therefore, I argue that the narratives of homeland shifted from abandoned ancestral homeland to multiple homes, which “myth of return” fails to explain.

(…) before I started my PhD, I had two options. My auntie is an MP in Cyprus, as I am a barrister, I had an offer from her to go and do some legal work for her in Cyprus, work with some NGOs, etc. I thought it would be my interest. So, I went, God many years ago, it must be 2008. I went to Cyprus to decide if I could live there for a little while. I went for a week, saw my grandparents, looked at flats, talked to my auntie about what the job might be like. And decided ‘I can't live here!' (Laughing). (…) So that was the only moment I think where I considered it but thought I could not do it. I don't have any aspiration to be there. I’d rather live in the United States. (Meltem, 30, Third-generation, First Wave, Turkish Cypriot)

Meltem told me some of her family members reside in Thailand and other parts of the world and that she travels around the world to visit them. Considering these aspects, her identity could be defined as a global elite or cosmopolitan rather than the diasporic identity that can only be completed with return to the homeland. As Bhabha (1994) argues, cultural encounters result in hybridity of cultural identities especially for transnational communities. For participants like Mary and Meltem who have the cultural capital to adapt to various culturalscapes, multiple identities in one’s self are not in conflict and one does not lose an identity when adapting another. Diaspora space is *third space* where cultures interact and are hybridized where an *in-betweens* identity is constructed (Bhabha, 1994). Based on this analysis, I suggest that female participants with formal qualifications and competency in English like Mary and Meltem, displayed a stronger sense of belonging to London as their adopted home compared to female participants with no qualifications. Although these participants came to the United Kingdom with the first wave of Turkish migration, a similar attitude is displayed by participants from later waves as well. Among the male participants more patriotic sentiments were displayed toward Turkey or Cyprus even among the more cosmopolitan or elite subjects. It suggests an ownership of motherland at the discourse level. According to this discourse, Turkey or Cyprus is the motherland that belongs to them although they have no plans of returning there. The alleged and felt symbiosis between a certain piece of Earth and its community Smith (1986) argues was displayed more rigorously by male participants.

After spending decades in London and adopting it as their new home returning to their country of origin is not an easy process even for first-generation migrants who planned to stay in the United Kingdom for a limited period (see Smith and Guarnizo, 1998). Mustafa narrated some of those first-generation migrants who attempted to return Cyprus ended up coming back to London.

Some of us had returned, but most of them came back here because they are used to living here. Also, in Cyprus, everybody knows each other and talks about who did what, it is a small place. And here it is better both socially and economically. So, we stopped talking about returning. We know it is not going to happen. (Mustafa, 83, First-generation, First Wave, Turkish Cypriot)

Even though myth of return is very commonly referred to in diaspora literature, most of my research participants told me that they do not have any plan or desire to move to Turkey or Cyprus:

All my family from my mother’s side live in London. I don’t think any of them are planning to go back to Turkey. We know we belong here. We are used to the system and lifestyle here. (Efe, 26, Third-generation, First Wave, Turkish)

As my research findings show, most of the members of the Turkish-speaking community decided to stay over in the United Kingdom and made it their permanent home. This is both because their country of origin has changed since they left and they have become accustomed to the cosmopolitan cultural environment of London. Also, encounters with foreign cultures and the *zeitgeist* of the postmodern and global era broke the barriers of closed identities that are rooted in a promised land which could only be achieved by returning to the ancestral homeland (Hall, 1990). Yet, I do not argue that the myth of return is a completely invalid concept and every community is cosmopolitan in the globalized world. However, I argue that the classical diaspora definition as it refers to Jews, Armenians and Greeks cannot be applied to most contemporary migrant communities to analyze narratives of homeland. In that sense, my research provides a major contribution to the field by suggesting a contemporary interpretation of the myth of return and sense of belonging among the Turkish-speaking communities in London.

During the interviews, none of my participants told me about ongoing discussions and/or plans about moving back to Turkey or Cyprus. My research findings suggest that the narratives are shifted from dreams and plans to return to homeland toward regular visits to their countries of origin for holidays. Hence, I argue that the “myth of return” among the Turkish-speaking community in London is transformed into short-term, annual returns during vacations. What Khan (1977) calls ‘institutions of migration’ - travel agencies, connect the diaspora with the homeland. Immigrants regularly go to their countries of origin for a vacation. According to Williams et al. (2000), these episodic holidays act as a stepping stone to a permanent return but merely play out as an illusion of return. However, I argue these visits have an alleviating impact on their desire to return (also see Khan 1977; Ali and Holden 2006). By providing these services, institutions of migration do not only benefit from the ethnic economy, but they also transform the diaspora. Because the narrative is shifted among the diaspora, nowadays institutions of migration promote summer houses for sale in Turkey or Cyprus enabling diasporans to have multiple homes.

Many immigrants keep their connection with both home and host countries, socially, culturally, economically and politically rather than breaking their attachment to one for the other. Some migrants take an active part in homeland politics, economy and religious life while others are highly involved in the country of settlement and engage in certain transnational activities such as economic investments (see Levitt, 2003). There are various levels of cross-border engagement and home-host mobility. For instance, there are some members of the community that consult and/or follow religious leaders in their country of origin closely. They are highly involved in the home country’s religioscape (spiritual life). Also, some members of the community travel regularly while some of them go back and forth and/or engage in transnational business while living abroad. Also, as my research participants narrated, many of the members of the community have properties in hometowns or villages which they originally invested in as part of a plan to settle down in Turkey or Cyprus when they retire. In that way, they keep their feet in both home and host countries. Among my research participants, eight of them owned properties in Turkey or Cyprus. However, as the narration of the myth of return fades away, owning properties serve the purpose of creating a sense of belonging to home countries for younger generations. The importance of homeland visits to understand Turkish, Kurdish or Cypriot culture better and transmit it to younger generations is emphasized by all fourteen first generation participants.

We always encourage our members (Kurdish Community Centre) to take their children with them when visiting Turkey and show them their villages and not to cut that link with their land. Also, we encourage people to buy a house in their villages even though they only visit once a year. Therefore, at least their children would know that soil and country belong to them. We want them to keep these links with Kurdistan, and visit it to teach our culture, customs and tradition to their children. (Mahir, 57, First-generation, Third Wave, Kurdish)

Although, Turkish, Kurdish or Cypriot culture is reproduced in London, most members of the community believe that the country of origin is where their culture is lived authentically. Annual visits to their home country have the purpose of seeing the extended family as well as teaching the culture and customs to younger generations. Mahir, a politically active member of the Kurdish community in London, spoke of the political importance of visiting Turkey. He is concerned not only about younger generations’ cultural identity but also linking Kurdish identity to land or soil. As a diaspora, he does not wish to leave his homeland behind and wants to keep the right to live there one day. However, he has not moved there yet, and defers his return to an unknown date. For Kurdish diaspora, having properties in Kurdish regions of Turkey is significantly important as the Kurdish nation building process is ongoing and Kurdistan’s borders are not yet set. Therefore, having a house which they will visit once a year is marking the Kurdish land as their own. However, this political action refers to a display of solidarity with fellow Kurds in the homeland or belonging to Kurdish nationhood rather than a myth of return.

The tourism pattern of the Turkish-speaking community is based on visiting Turkey or Cyprus in every *izin* (vacation). As my research findings suggest, many of the Turkish-speaking immigrants, especially those from the first-generation, maintain their sentimental links with their home country by regular visits. 15 of my research participants, 11 of whom are first-generation migrants, narrated the importance to them of visiting Turkey or Cyprus regularly. Baldassar (2001) coined the term “return visits” to describe the migration experience of Italian diaspora in Australia to the country of origin. What Levitt (2003) calls “roots journeys” refers to visiting the ancestral homeland and becoming reacquainted with relatives. On the other hand, home country visits are not limited to visiting family and relatives. There are some pragmatic reasons such as going to holiday resorts and historical sites for pleasure where the food, music and culture is familiar to their taste or for the purchasing of goods and use of cheaper services such as private health or dental services.

Well, I go there 3–4 times a year. But I don’t usually go to the village and stay for that long. I know from previous experience how boring it gets. So, maximum I go for like a few days and then to holiday like beach somewhere and then I come back. I prefer to go there for my holidays because I love going to Turkey. I love the beach, I love the weather, I love the food and the people. So that makes me enjoy my holiday basically. I have been to other holiday destinations, but it just doesn't feel the same. I went to Spain and France. I didn’t like it. So, I always prefer to go back home to Turkey for a holiday. (Gizem, 30, Second-generation, Second Wave, Kurdish)

Second and third generation participants approach visits to Turkey or Cyprus pragmatically. Gizem and Alice explained that they only visit Turkey as a holiday destination where they are familiar with the culture and food from their annual visits. I argue that these visits cannot be classified as what Baldassar (2001) and King, et al. (2000) call “return visits” because visitors do not attach sentimental meanings in the same way first-generation migrants do.

Some of the community members are involved in transnational jobs that include visiting their home country to buy or sell goods and products. Some of them bring cultural products from their home country and sell them in London. Items brought back include food, drinks, clothes, ornaments and liturgical objects.

So, I grew up in the culture, my father was one of the first to import Turkish music, cassettes, books, *tespihs* (prayer beads), circumcision suits (laughing) all of the kind of stuff that culturally originated from Turkey. (Ekrem, 57, Second-generation, First Wave, Turkish Cypriot).

Ekrem told me about the influence of Turkish music on him as he was growing up. He is from the first wave and so during his childhood there was not the vibrant Turkish culture in London as it is today nor satellite TV to watch Turkish soap operas. Therefore, these imported cultural products played a significant role in construction of identity for the community.

Even though some Turkish-speaking migrants left Turkey or Cyprus 20–40 years ago, they are not forgotten by their contemporaries and they update themselves about life in the homeland by visiting or by asking anyone who has visited it recently. Those who visit update others about what is going on at *memleket* and in that way they keep the narratives about home alive.

Some of the immigrants visit their countries of origin for marriage purposes. Some young members of the community visit their home country and find partners themselves while for some others, parents make arrangements for their children and find potential partners from their hometowns or villages. I argue, this kind of ethnic endogamy aims to ensure a continued reproduction of identity and tradition (see Böcker, 1994).

## Return Visits: Homeland Visiting Patterns

Some of the members of the Turkish-speaking community save their money and go to their home country for a summer vacation every year almost as a ritual (Mehmet, Begum, Guler, Turkan, Yasar, Ela, Emel, Kemal). Turkey or Cyprus is also the place where they forget mundane worries of diasporic life and return to themselves to enjoy sweet memories of the past. Based on Eliade, (1987) and Margry, (2008) argument, I argue that these ritual-like visits are *mundane pilgrimages*. Margry, (2008) states that those taking a pilgrimage seek an encounter with a particular cult object at the shrine in order to acquire spiritual, emotional or physical healing benefits. In my research case, migrants visit their homeland to escape a profane environment of diaspora to gain the spiritual and nostalgic experience of memories in their ancestral land and either heal their identity crisis or help the identity construction of younger generations. Therefore, I argue that Turkey or Cyprus are sanctified with the nostalgia of the past and images representing or reminding cultural identity. The pilgrims of diaspora travel not for the sake of heaven or other transcendental benefits but to find assistance with their existential or identity questions by linking their identity to collective memory and ancestral homeland. Visiting relatives has more meaning than simply keeping social networks; it strengthens a sense of belonging to the nationhood. In this dichotomous relation, Turkey or Cyprus with the symbols and memories it carries represent the sacred, while diaspora life in London is involved with mundane individual concerns.

Such a feeling to visit Turkey! [Euphorically] Let me tell you an anecdote, a Turkish man living in Germany sees a car parked on the street that came from Turkey. He removes the cap from the tyre valve, starts to deflate the car’s tyre and inhales the air. The owner of the car sees him and asks what are you doing, hemşerim (fellow villageman)? The man responds I am taking the smell of *memleket* (homeland). Our case is similar, when I visit Turkey, I feel blessed. Going to the *memleket* is special for me! (Yasar, 54, First-generation, Second Wave, Turkish).

Yasar was very enthusiastic and euphoric when he was talking about his visits to Turkey. His quote summarizes conservative Turkish economic migrants’ romantic views of Turkey. According to that view, even Turkey’s air and soil is idealized and breathing its air or touching its soil metaphorically refers to reuniting with Turkey. Therefore, they visit Turkey on every *izin* (vacation) to satisfy their desire for Turkey and its culture. Throughout our conversations in the interviews I asked my participants about their home country visits and mapped out their visiting patterns as follows:

*Frequent travelers*: those still firmly tied to the homeland. They have land or property in their hometown or village and go back and forth between home and host countries regularly. *Periodic travelers* visit their home country for the same period of the year (annually and generally summer time) as a duty or a kind of profane pilgrimage. *Intermittent travelers* are those immigrants whose lives are rooted mainly in the host country and visit their home country sporadically. However, they keep contact with people in the homeland and track the life of the community in the homeland by asking reports from those who visited it recently. However, they do not have enough time, money or enthusiasm to visit their home country frequently. *Fugitives* are those people who have escaped from their home country for various reasons and have taken refuge in the United Kingdom. Their situation is precarious, and they are not allowed to visit Turkey or Cyprus for a certain period (until they have a residence permit from the British authorities) or even in their lifetime (in cases where they are convicted by the Turkish authorities for political or violent crimes). Any member of the Turkish-speaking community, from any generation, wave or ethnic group, could be a part of any of these categories as this classification is based on the practice of traveling and performativity rather than an ascribed status.

### Frequent Travelers

Frequent travelers visit the homeland more than once a year for business or for a holiday. However, they tend to stay for a shorter period in comparison to periodic travelers. Their visits are generally no longer than a week. They are mostly those with financial security and legal resident status in both countries that enables them to travel without any problem. They tend to visit the touristic places of their home country as well as the financial centers rather than visiting their home towns or villages.

I go to Turkey many times throughout the year for a few days for business. Also, when we have a break from work, we talk about going to some European countries. But at the end of the day, we say come on what are we gonna do in Germany or Belgium? Let’s go to Istanbul for three days and chill. (Efe, 26, Third-generation, First Wave, Turkish)

Frequent travelers visit Turkey or Cyprus often because they are familiar with it and like its culture. However, Efe’s approach to Turkey is not a romanticized view. He explained that it is cheaper to go on a holiday to Turkey, and that he does not need to worry about getting lost or finding food for his acquired taste. Although, these regular visits can be defined as “return visits” (see Baldassar 2001), they do not act as a stepping stone to a permanent return. Instead, these episodic visits and narrations of visits replace the myth of return.

### Periodic travelers

Visiting their homeland in every *izin* (vacation) was also frequently mentioned by my participants. At many gatherings of Turkish-speaking people, the theme of traveling to Turkey or Cyprus emerges. At these gatherings, recent trips are discussed, and future trips are planned or dreamed about (see Mandel, 1990). The key timings of such periodical visits are during summer vacations or on religious days such as Eid. The duration of these periodic visits varies from two weeks to two months.

In July you cannot find anybody here (North London) for 4–6 weeks until the schools start. Everybody goes to Turkey. (Yasar, 54, First-generation, Second Wave, Turkish).

As Yasar told me many of the members of the Turkish-speaking community visit Turkey or Cyprus annually. Especially for economic migrants, it is something they look forward to and save up for throughout the whole year.

Many of the first-generation migrants emphasized the importance of visits to their home country to catch up with both family and the friends they left behind as well as creating a sense of belonging that can be transmitted to younger generations. In that way, first-generation migrants aim to form younger generations’ opinions toward the home country and transmit Turkish, Kurdish or Cypriot identity to them. Families arrange regular contact with their home country to ensure that their children reproduce their national identity or get to know their fellow compatriots in the homeland. There are two motives behind this practice; first introducing children to family in the homeland and strengthening their bonds with their compatriots. Secondly, visiting elderly members of the family and relatives is a custom in Turkish tradition, especially in rural parts of Turkey. With visits to their ancestral homeland they become reacquainted with relatives, thus these visits can also be called “roots journeys” (see Levitt, 2003).

Moreover, the Turkish-speaking migrants save money to go to Turkey or Cyprus for summer vacations almost every year in a ritualistic fashion. Turkey or Cyprus is also the place where they forget the stress of business life from living in a foreign culturalscape and they return to themselves hence why I refer to these visits as “*mundane pilgrimages*” (see Eliade, 1987; Margry, 2008).

As Begum said, first-generation migrants attribute a sentimental meaning to visits to their home country, such as bridging the gap between younger generations and their ancestors. In this way, they aim to strengthen the collective memory and reproduce cultural identity. However, the frequency and duration of visits to the homeland decrease as their children grow older.

When we were kids, as soon as it was the summer holiday, we were on the plane to Cyprus and we came back 31^st^ of August as we went back to school on first of September. Now it's like a week, ten days that's it. See the grandparents, aunties, uncles, cousins, go to the beach, and come home. (Meltem, 30, Third-generation, First Wave, Turkish Cypriot).

Eleven research participants who are the first-generation immigrants mentioned having a house in their hometown village or in a holiday resort that they stayed in during periodic visits, coupled with a vague plan of settling down there in the future. They build houses in their home country, leading to an ostentatious reputation of showing off the wealth they have gained through their diasporic experience (for a similar study about Bangladeshi community see Gardner and Ahmed, 2006).

### Intermittent travelers

Some members of the Turkish-speaking community visit their country of origin less frequently, such as once every few years. One of the most common themes in the interview analysis was the young generations’ lack of interest with homeland visits, as was narrated by the first-generation immigrants. Also, the second and third generation members of the community frequently described their hometowns or villages as the place that their parents or grandparents came from rather than their own homeland. Almost all of them described hometowns or villages as uninteresting and they said that they only visit them once in a few years to see relatives out of family necessity.

(…) maybe once of every two years, we visit because we have elderly relatives in the North [Cyprus], so we go there. The last time I went was two years ago. (Alice, 27, Third-generation, First Wave, Turkish Cypriot).

Dessí, (2008), Rothstein (2000) and Noriel (1995) argue that collective memory still induces a common social behavior. However, my findings challenge their argument and demonstrate that the influence of collective memory on behaviors of younger generations is very limited. Visits to Turkey are not frequent among the second and third generation. Almost all of the younger generations described their parents’ home towns or villages as unattractive and uninteresting. First-generation migrants desire to visit their home country is rooted in childhood or youth memories whereas the second and third generations have stories of their parents in their collective memory, yet their first-hand experiences contradict those stories. Therefore, when they age, they lose interest with their parents’ hometown villages.

### Fugitives

As Kunz, (1981) theory argues, in any refugee wave, people experience different encounters in the host country based on their marginality within or identification with their former home county. Kunz classifies displaced communities under three categories. The first group consists of majority-identified who identify themselves enthusiastically with the nation they have left behind. The second group consists of event-alienated refugees who are ambivalent or embittered in their attitude to their former compatriots, such as religious or ethnic minorities who have been marginalized or discriminated against by the majority population of their country of origin. The third group consists of self-alienated refugees who for various ideological reasons have no wish to identify themselves with their home nation (see, Al-Rasheed, 1994; Weiner, 1996; Bloch, 2002).

In some cases, immigrants are not allowed to visit their home country for years due to political reasons, legal status issues, or an on-going civil war at their country of origin. All of the political refugees narrated not being able to visit Turkey or Cyprus for years after they came to the United Kingdom either because of ongoing cases/trials, convictions due to political activities or because of ambiguity in their legal status in the United Kingdom as asylum seekers. This final reason was very common especially among Kurdish and Alevi members of the community. On the other hand, Turkish Cypriots suffered an ethnic cleansing and civil war in Cyprus and they could not visit their home country during troubles. Furthermore, Cyprus is now divided following the civil war which has resulted in Greeks living in the south of the island and Turks living in the North. Those Turkish Cypriots who used to live in the south of the island were required to leave their hometowns or villages, and they were not permitted to return for a visit for decades. My research findings suggest that those who are not allowed to visit their home countries romanticize it more significantly. Mahir, a Kurdish activist, narrates when he goes to bed every night he thinks of his hometown in the Kurdish region of Turkey. When he was talking about his only visit to Turkey after 24 years his eyes lit up with joy and excitement. Also, some of the political refugees interviewed escaped from Turkey or Cyprus as they were convicted of political crimes and were not allowed to visit their home countries for decades:

I got my British passport very quickly however I couldn’t go to Turkey for 11 years because of ongoing trials over there. (…) My mum died when I was here, and I visited her grave ten years later … (Kazım, 54, First-generation, Second Wave, Turkish)

When narrating his story Kazım’s eyes were brimming with tears; he took a big sip of his beer and walked away leaving the *kahvehane* (Turkish coffeehouse) to cry outside. Although participants in this group fits Kunz (1981)’s second category of refugees who are event alienated, as the examples of Mahir, Kazım and Rojda suggest, politically active members of the community, especially those who are persecuted in Turkey for their activism, display a stronger sense of belonging to and romanticized view of Turkey as a homeland. This applies to first- and second-generation migrants from any wave of migration and any gender. Therefore, their myths refer to return to an imagined version of the homeland.

In addition to these four-categories of homeland visiting patterns, there are some elderly retired members of the community who live three to six months in Turkey or Cyprus and spend the rest of the year in the United Kingdom. In that way, they both appease their longing for their homeland and keep up with their children and grandchildren living in the United Kingdom.

Some people among the first-generation experienced significant trauma during the civil war in Cyprus or Turkey, and they have not visited their home country since they left. Also, there are some exceptional cases of second or third-generation “Turkish origin” people (mostly children of mixed marriages) who have never been to Turkey or Cyprus in their entire lives. These are mostly people who weakly identify themselves as Turkish because they do not see Turkey or Cyprus as their homeland but as the ancestral land of one of their parents.

In both Alice’s and Bob’s cases, they share the traumatic experience of a civil war in their collective memory. They associate Cyprus with this trauma which they want to leave behind. Alice’s grandmother has not been back to Cyprus after they came to the United Kingdom and Bob’s grandparents do not talk about Cyprus at all. He did not even know which city his mother was born in until her death.

### Post-mortem Travel of Body: Another Form of Myth of Return “Take Me Back to Homeland Dead or Alive!”

As I have discussed above, the “myth of return” or in other words desire to settle down in home countries has been transformed into episodic holidays that play out an illusion of return (Williams et al., 2000). However, some sentimental links with the country of origin or family living there were still frequently articulated by the research participants. This feeling is referred to as *özlem* which means longing in Turkish.

I live in England missing my country, my family. I wake up some nights missing my country, my land. (Mahir, 57, First-generation, Third Wave, Kurdish).

Elif, a second-generation woman who spent her childhood in London, finds her parents’ hometown dull and she does not want to visit Turkey every summer. Yet she feels *özlem* for Turkey. I argue that this feeling of *özlem* toward a place that she does not desire to visit or return to is a *placebo nostalgia* (Poupazis, 2014) which stems from collective memory. However, Mahir’s feeling of *özlem* is a combination of romanticized and politicized views about his lost homeland. He is missing the Kurdish land and the imagined Kurdistan he fought for.

As I argued before, the narration of the myth of return has shifted. In addition to the narrations of return visits or mundane pilgrimages, some participants narrated their desires to be buried in their hometown or villages when they die. Most of the first-generation research participants expressed their wish to return to their country of origin before they die or to be buried there after they die.

Even though I am British citizen, I belong there (Turkey). Unless I go there alive, only Allah knows what will happen tomorrow; my body will definitely go there, dead or alive. (Turkan, 51, First-generation, Second Wave, Turkish)

I testified to my children to bury me in Kurdistan when I die. I cannot detach from that land. (Mahir, 57, First-generation, Third Wave, Kurdish)

Transferring a body from the United Kingdom to Turkey is an expensive process, and there is not any religious requirement to do so. In Britain, designated areas in cemeteries are allocated for Muslims, and there are not any restrictions on Muslims being buried in non-Muslim cemeteries. Also, in the Islamic faith, location does not have any bearing in sending prayer and so people can pray from Turkey or Cyprus for those loved ones buried abroad. However, for the community members, being buried in their homeland has a symbolic value. I argue that this narrative replaced the myth of return and it is perceived as a return to their roots or as a display of belonging. In addition, some members of the community see burying bodies in the homeland as a last duty toward their beloved ones. For them, transferring deceased bodies seems to meet the ultimate wish of every migrant subject: returning to their homeland. This argument is another original contribution of my research to the diaspora literature, as it has never before been discussed in depth.

Well, some people bury their deceased family members here. It is okay, in most of the Christian cemeteries, there is a section allocated for the Muslims. But being buried in a Muslim country is different. I went on pilgrimage to Mecca, but I don't even want to be buried over there. I want to be buried in my homeland. Would you not want to be buried in Turkey? Your relatives would pray for you when they visit the cemetery. The cemetery is at the centre of our village, so when people pass by, they can pray for you. We say take me back to the homeland dead or alive, you know. We don’t want even our dead bodies to stay over here for eternity. (Yasar, 54, First-generation, Second Wave, Turkish)

As a response to these demands, the Turkish Religious Foundation (TRF) created the funeral services solidarity fund to help Turkish citizens living abroad to repatriate the body of their family member to their village. The TRF’s United Kingdom branch offers the same service to Turkish-speaking people living in diaspora. This fund works as a form of insurance where people pay annually for themselves or their family and when they pass away; the Turkish religious Foundation covers all the funeral expenses and sends the deceased to their hometown or village. Yasar expressed his delight for this service:

The expense of a funeral in London is around £3,000. If you transfer the body to Turkey, it costs around £5,000. But if you register this fund, you annually pay small amounts of money. And when you die, they start your funeral proceedings here like registration with the Turkish Consulate, washing up the body, doing the prayer at the Turkish mosque, and then transferring the body with an attendant to wherever you want to be buried in Turkey. (Yasar, 54, First-generation, Second Wave, Turkish)

After narrating this enthusiastically Yasar also shared the brochure of the TRF’s funeral fund with me in the event I was interested in doing the same. As I argued previously, Yasar’s interview summarizes conservative Turkish economic migrants’ views of Turkey which romanticizes and idealizes even inanimate objects such as Turkey’s air and soil as the sacred homeland. This view is closely intertwined with their social class. Members of this group do not have formal qualifications recognized in the United Kingdom and their competency in English is limited. As a result, they are working class members of the community working or managing kebab shops, off-licenses and dry cleaners. Their loyalty lies with their homeland; they place themselves in Britain as temporary economic migrants who will reunite with their roots one day. Unlike other members of the community who display more cosmopolitan identities, London has not been a welcoming home for them. This strong sense of belonging to the romanticized homeland is displayed by members of the community regardless of their migration wave and gender. However, it is only limited to first generation migrants as for the following generations socializing and gaining qualifications in the United Kingdom brings a sense of upward social mobility and attachment to London as a sense of home. A lack of nostalgic experiences such as a childhood and upbringing in Turkey chips away at the meaning of homeland visits.

## Concluding Remarks

In this article, I discussed North London’s Turkish-speaking community’s narratives of homeland as well as their transnational mobility after they settled in the United Kingdom. I challenged the diaspora literature’s overgeneralizing portrayal of migrants as people who are motivated with the idea of return and whom always struggle to maintain links with their homeland. I comprehensively discussed the current phase of the “myth of return” among the community and how it has transformed into episodic homeland visits in reference to cultural, socio-economic and ethnic backgrounds of the members of the Turkish-speaking communities in London. I also contributed to the literature by discussing how the procrastination of return migration has evolved into the wish to be buried in their hometown village. I also expanded on transnationalism by analyzing community members’ homeland visiting patterns in four categories as well as analyzing how identity is (not) tied to a place of origin and how different interpretation of the myth of return reproduced their identities.

## Data Availability

The original contributions presented in the study are included in the article/Supplementary Material, further inquiries can be directed to the corresponding author.
